# Feasibility of clinical EEG for music recognition in children aged 1–12 years

**DOI:** 10.3389/fped.2024.1427118

**Published:** 2024-10-11

**Authors:** Janeen Bower, Sebastian John Corlette, Mengmeng Wang, Wendy L. Magee, Cathy Catroppa, Felicity Anne Baker

**Affiliations:** ^1^ Faculty of Fine Arts and Music, The University of Melbourne, Melbourne, VIC, Australia; ^2^ Department of Music Therapy, The Royal Children's Hospital Melbourne, Melbourne, VIC, Australia; ^3^ Clinical Sciences, Murdoch Children's Research Institute, Melbourne, VIC, Australia; ^4^ Department of Anaesthesia and Pain Management, The Royal Children's Hospital Melbourne, Melbourne, VIC, Australia; ^5^ Department of Paediatrics, The University of Melbourne, Melbourne, VIC, Australia; ^6^ Department of Biomedical Engineering, The University of Melbourne, Melbourne, VIC, Australia; ^7^ Boyer College of Music and Dance, Temple University, Philadelphia, PA, United States; ^8^ Melbourne School of Psychological Sciences, The University of Melbourne, Melbourne, VIC, Australia

**Keywords:** music, EEG, pediatrics, feasibilities studies, GED (generalized eigendecomposition)

## Abstract

**Introduction:**

Musicality is an innate capability and the fundamental architectures necessary for music processing are present from birth. However, there is a notable gap in pediatric specific music neuroscience research and research that employs ecologically valid musical stimuli.

**Methods:**

This pragmatic feasibility study aimed to assess the utility of EEG collected via pre-existing clinical monitoring to describe the processing of familiar song as an ecologically valid stimulus, in the underrepresented pediatric population. Three comparative auditory conditions (song, speech, and noise) were utilized to assess the changes in EEG across these conditions compared to a baseline silence.

**Results:**

Analysis of EEG data from a pilot sample of four children revealed distinct changes in the underlying frequency components of the EEG during the song condition that were not observed in either the speech or noise conditions. To extend this analysis, a uniquely hypothesis-driven, multivariate statistical analysis method (generalized eigendecomposition [GED]) was employed, however in this study we did not isolate a consistent source responsible for the observed changes in the frequency components of the EEG during the song condition.

**Discussion:**

The study is limited by the small sample size but nevertheless demonstrated feasibility of collecting EEG data in the imperfect auditory environment of an acute clinical setting to describe a response to an ecologically valid stimulus in the underrepresented pediatric population. Further research with a more restrictive study design and greater participant numbers is needed to extend these preliminary findings.

## Feasibility of clinical EEG for music recognition in children aged 1–12 years

As a complex auditory stimulus, music consists of many components including melody, rhythm, harmony, and timbre. While these components may be processed separately, music is experienced as a rich phenomenological “whole” (1–5). Publications that objectively describe the neural processing of music overwhelmingly include adult participants aged 18 + years (6). The brain of a child is different to that of an adult in size, shape, tissue composition, and functional connectivity (7). Further, experience dependent neuroplastic changes, and structural and functional connectivity are rapidly emerging during childhood (8). Therefore, care should be applied in translating the current music neuroscience evidence directly to children.

Engaging in music is an inherently human experience (9). Emerging neuroscience research that has explored components of the auditory evoked potential (AEP) suggests that term neonates display sensitivity to pitch, beat, duration, and tonality (10, 11). Further, functional magnetic resonance imaging (fMRI) research in neonates indicates that music stimulates a hemodynamic response throughout a bilateral network of cortical and sub-cortical regions (10, 12–15). Thus, the primary architectures of music perception appear to be present from birth. Passive musical enculturation throughout the early childhood years then results in the development of complex musical knowledge, including an implicit understanding of Western musical syntax such as the pre-conscious processing of melodic and harmonic expectations (16).

A recent systematic review describing music processing in neurotypically developing children, from term birth to 18 years, identified a significant gap in evidence in the age cohort 1–18 years (6). Twenty-three of the 46 included studies explored the neural processing of music in infants aged less than one year. This is likely because imaging occurred when the infants were in a natural sleep state. Sleep state imaging increases the quality of data by reducing movement artifacts. This control of state is difficult to implement in older infants and young children (17). Inattention and emerging language skills result in a reduced ability for infants and young children to follow instructions and remain motionless for extended periods of time. This is particularly relevant when measuring hemodynamic responses captured by fMRI which, in addition to poor temporal resolution, require the child to stay still for data acquisition with high spatial resolution (17). Electroencephalography (EEG) may therefore provide a more practical methodology to explore music processing in young children due to the high temporal resolution and tolerability for subject movement (18). Further, scalp EEG is non-invasive and the associated equipment is portable which increases the feasibility of this scanning methodology relevant to the complexities of the pediatric population (18).

Current EEG research describing components of the AEP has offered emerging insight into specific neural processes in infants and children relating to music processing and the localization of this processing. This has included sensory functions of pre-attentive auditory memory measured with the mismatch negativity (MMN) component of the AEP and more complex cognitive processing of Western music syntax measured with the early right anterior negativity (ERAN) (11, 19, 20). A significant difficulty in connecting this AEP research to developmental or clinical populations relates to the scientific reductionism employed with the use of highly deconstructed musical elements used as the auditory stimulus. For example, the mismatch negativity (MMN) utilizes an oddball paradigm to detect a deviation in a musical element against an internal memory representation. When exploring a pitch induced MMN, the oddball paradigm typically includes the rapid and frequent presentation of a single tone “standard” with an infrequent pitch “deviant” (21). While AEP and its components are valuable for describing pre-attentive auditory and cognitive processes, they alone are insufficient to understand the neural processing of music (22). The stimuli used to measure these components of the AEP are isolated from the richer “whole” music experience that is encountered in everyday life.

The scientific reductionism of music utilized in auditory science research also reduces the ecological validity of the evidence related to therapeutic applications of music. For the purpose of this study, “whole” music is defined as music that combines the basic components of melody, harmony, timbre, and rhythm, and is organized according to the rules of Western musical syntax. Hemodynamic methods of brain imaging have successfully described brain responses to whole musical experiences in infants (13); however, as described, these methods are difficult to implement in children and necessitate data collection in foreign non-naturalistic settings. Research with adults has begun to address this gap related to the naturalistic stimulus and setting (23–25).

Pediatric specific research that explores the neural processing of a whole music is needed to expand our understanding of the developmental trajectory of music processing relevant to cortical maturation and to extend theoretical foundations and mechanistic understandings of music-based interventions for children in clinical settings (e.g., music therapy or music medicine interventions). Hence, with consideration given to the paucity of pediatric specific music neuroscience research, and the complexities of undertaking this research, the purpose of this study was twofold. Firstly, to explore the feasibility of acquiring EEG data via the pre-existing clinical equipment in an acute healthcare context that are sufficient to describe a difference in EEG response to familiar song and comparable controlled auditory stimuli, should such a difference exist. Secondly, to explore a novel method of hypothesis driven multivariate EEG analysis that considers that the generators of EEG signals responsible for processing music likely span many channels of the EEG (26). The null hypothesis addressed by this multivariate EEG analysis was: *Familiar song does not generate a unique and reproducible brain response, compared to speech, noise, and silence, as measured by EEG*.

## Method

This prospective, single site study was undertaken at The Royal Children's Hospital Melbourne (RCH) Australia. Ethics approval and governance authorisation were granted by the Human Research and Ethics Committee at RCH (HREC #74146) to recruit 10 participants. Pragmatic decisions were made to recruit children who had a clinical indication for EEG monitoring but did not present with status epilepticus or were diagnosed with a significant brain disease or disorder. The advantage of this design was the opportunity to collect high quality EEG data via the clinical systems even within the imperfect auditory environment of an acute healthcare setting. The beneficence vs. burden of this research in children was also a significant consideration in this design because the application of EEG leads in a non-clinical population is potentially of high burden for the children and their families/guardians which likely contributes to low recruitment and underrepresentation of the pediatric cohort. The *n* = 10 was a further pragmatic decision, chosen in consultation with the Neurology Team and anticipated number of patients likely to be suitable for participation during the study period.

Convenience sampling was employed within the Neurology Department at RCH between August 2021—May 2022 of children undertaking either inpatient video-EEG monitoring or ambulatory EEG (only) monitoring.

### Inclusion criteria

Inclusion criteria were intentionally broad to maximize potential recruitment. Children aged 1–12 years, who had English as their dominant language, and had non-emergency EEG monitoring at the RCH were approached for recruitment. Given the recruitment of children, participants were required to have a legally acceptable representative capable of providing informed written consent for participation.

Children who were diagnosed with a severe neurological illness or injury, or a neurodegenerative condition were excluded from participation. This included children with low functioning autism spectrum disorders, cerebral palsy, severe developmental delay, acquired brain injury, etc. Children with diagnosed hearing loss were also excluded as the experimental protocol required that the child listen to comparative auditory conditions.

### Screening

Prior to recruitment to this study, the EEG of potential participants was visually assessed by the Neurology Team at RCH to observe gross abnormalities, including frequent interictal epileptiform discharges, electrographic seizures, period patterns and/or significant background abnormalities that may have impacted the collection of auditory responses. If such activity was present, the child was not approached for recruitment. Further, if EEG collected during the experimental session was visually assessed to contain frequent epileptic discharges or seizures, this data was excluded from analysis.

### Data collection

Diagnostic and demographic data were collected prior to the experimental session. EEG data collection occurred within the context of continuous clinical EEG monitoring. Twenty-three leads were applied according to the standard 10/20 format. This included two additional anterior temporal leads (T1 and T2) per the clinical protocol. Electrodes were 10 mm silver/silver chloride cup electrodes adhered with Ten20 conductive paste. Clinical EEG at RCH utilizes free lead placement instead of a cap given the improved impedance. Under the clinical protocol, all EEG leads were applied by expert EEG technicians. EEG was acquired via a Compumedics Siesta System Ambulatory Recording Device for inpatients, or Compumedics Grael V2 EEG for outpatients.

### The experimental session

**Figure 1:**
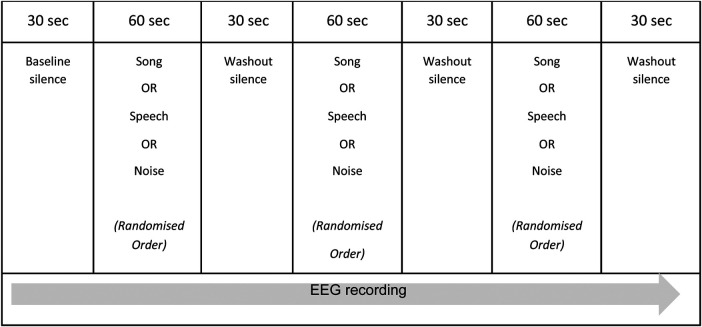
The experimental session.

The experimental session was pre-recorded and consisted of the presentation of the three comparative auditory conditions: song, speech, and white noise. These auditory conditions were presented in a random order and separated by short periods of washout silence, as outlined in Figure 1. Baseline and washout periods were included in the experimental session to control for any priming and/or habituation effects of the auditory conditions. The beginning of the experimental session was indicated via an audible “click” in the recording. The randomization of the auditory conditions was undertaken by an independent statistician and assigned each participant (*n* = 10) to one of six possible permutations of the three auditory conditions. R version 4.1.0 (R Foundation for Statistical Computing, Vienna, Austria) was used to generate the randomization. The total duration of the experimental session was five minutes.

The noted paucity of EEG research exploring brain state responses to whole song in the pediatric population means there is no direct precedent for the design of the experimental session. O'Kelly et al. (27) utilized a similar protocol when describing neurophysiological responses to various music conditions in adults presenting with profoundly impaired consciousness following brain injury, however their experimental protocol was approximately 30 min in duration. The complexities of undertaking neuroscience research with children related to short attention span, poor concentration, and/or reduced ability to follow directions were considered in the research design and supported the decision to maintain a short duration for the experimental session.

The experimental session occurred in either the child's hospital room or an outpatient EEG suite. The RCH has specifically equipped inpatient rooms for EEG monitoring which are single bed configuration, and the outpatient EEG suites are similarly specifically equipped and single seat. Single bed/seat configuration for EEG reduced the potential for extraneous noise however these rooms are not noise-attenuated and are situated in an acute hospital setting. Signs were placed on the doors to these rooms to ensure there was no unnecessary interruption during the experimental session. Auditory stimuli were presented to the participants via an mp3 player and noise cancelling headphones. The volume of each condition was adapted so that the average sound level of each condition through the headphones was approximately 60 decibels (± 2%). Each child participated in one (1) experimental session that included the three auditory conditions presented in a random order. To control for state during the presentation of the auditory conditions, each participant watched the same silent movie on an iPad. Similarly, recorded conditions were chosen over the live presentation of song and speech to further control state. The beginning of the experimental session (indicated via a “click” in the recording) was manually noted in the EEG via activation of an event marker to ensure temporal accuracy for data analysis.

The comparative auditory conditions presented in the experimental session were:

1. Song. The song condition was the pre-recorded presentation of the nursery rhyme “Twinkle Twinkle Little Star” with a simple arpeggiated acoustic guitar accompaniment. The song was sung by the first author (JB) who is an experienced music therapist, and was recorded using GarageBand Software (Apple Inc, Version 10.3.1). “Twinkle Twinkle Little Star” is a well-known Western nursery rhyme and was selected because of its cultural familiarity in the Australian context. While the song likely has different memory, emotional, social, and emotional meaning across various age cohorts, it is melodically and harmonically simple, repetitive, and follows the rules of Western musical syntax including resolution of harmonies at cadence points and thus the song was standardized across participants. The song condition had a duration of 60 s, and the song was repeated twice during this time.
2. Speech. The speech condition the pre-recorded speaking of the lyrics of “Twinkle Twinkle Little Star” with no musical or instrumental accompaniment. The lyrics were also spoken by the first author (JB) to control for any response to the timbre of JB's voice. The speech condition was also recorded using GarageBand Software (Apple Inc, Version 10.3.1). The lyrics from the familiar song condition were used in the speech condition to account for any potential memory or emotional connections the participants may have to the text. The lyrics were spoken in natural speaking voice and naturally retained some elements of rhythm, vocal timbre, and prosody. However, the speech condition did not include the wider pitch range present in the song condition, nor did it include the accompaniment of a harmonic instrument. Complete words and sentences were utilized, as opposed to the deconstructed auditory or speech stimuli, to increase the ecological validity of the condition. The lyrics were repeated three times for a total duration of 60 s.
3. Noise. White noise was used as a non-musical, non-language, auditory control condition of 60 s.

### Data analysis

Given the feasibility design of this study, all data analysis was undertaken per individual participant data. While a baseline silence was included at the beginning of the experimental session, upon inspection, this data included significant signal and external noise as the participants settled into the experimental session. Therefore, for the purpose of the following analysis, the silence data were drawn from the 30 s following the noise condition for each participant.

### Pre-processing

**Figure 2:**
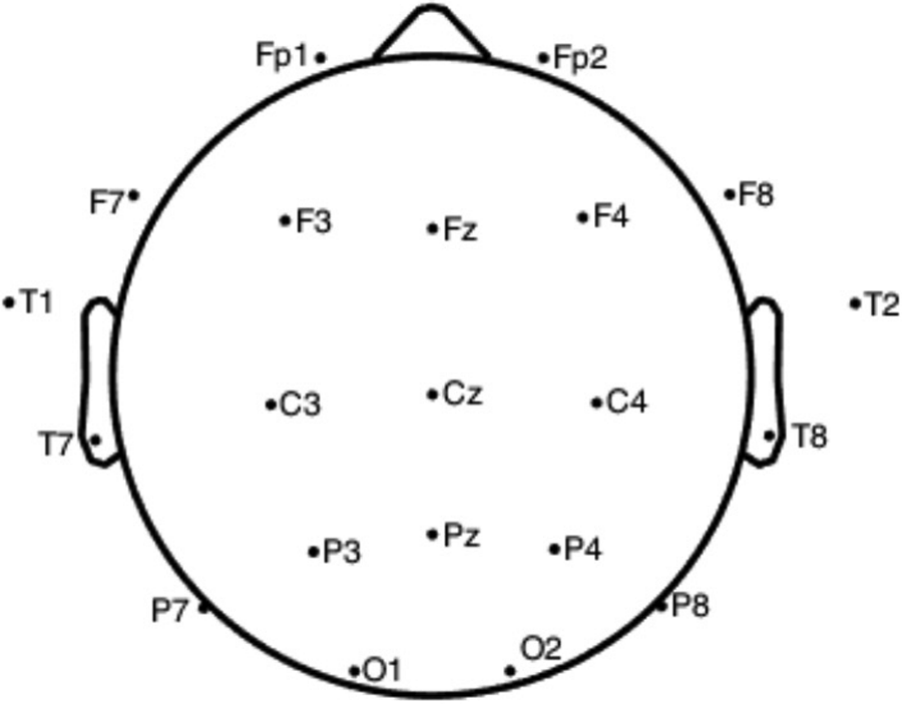
EEG channel locations (odd numbers = left/even numbers = right).

EEG data were trimmed to 30 s per condition to align with the duration of the silence condition. The 30 s epoch was taken from the middle of each auditory condition to control for any startle or priming effect of the condition onset and to ensure temporal accuracy of any analysis. Pre-processing of the data was undertaken in EEGLAB. Data were imported and standardized to the 21 channels that were common across all participants. Channel locations are included in Figure 2. Artifact rejection was completed visually and then using Independent Component Analysis (ICA) and independent components (ICs) of eye and muscle artifacts were then rejected. Data were then filtered utilising the FIR filter in EEGLAB with a frequency passband of 0.5–40 Hz and re-referenced to an average reference before analysis.

### Time domain signal analysis

The maximum difference in each channel for each condition over the course of 30 s was calculated and visually represented. The variance in each channel for each condition over the 30 s duration was also calculated and visually represented.

### Frequency analysis

Fast Fourier transform of each auditory condition was followed by inspection of the amplitudes of frequency content in the frequency spectrum. The mean amplitude for each channel across the broadband spectrum, plus the most common frequencies (delta, theta, alpha, and beta) was calculated. Frequency analysis results were represented using bar charts and topographic maps.

### Analysis of variance

Generalized eigendecomposition (GED) for the song condition vs. a baseline silence was implemented to extend the analysis of the response to the song condition. GED analysis was implemented as per Cohen (26). A pre-processing step, zero-phase component analysis, was undertaken prior to GED to improve spatial sensitivity of the data. Scree plots and component topographic maps were developed for the first five components for each participant. A common critique of EEG is the poor spatial localization of responses, and the inclusion of a GED analysis supports the goal of source separation and localization of complex cognitive tasks (for example music processing) via multivariate statistical analysis

Scalp voltage measurements reflect the spatial summation of the underlying electrical activity from the brain and the magnitudes of these voltages inherently vary over time and electrode locations. When voltage variations appear to be related in a deterministic way, they are said to co-vary, and the mutual variance between each pair of electrodes can be calculated and presented in the covariance matrix. Eigendecomposition is the mathematical analysis of this matrix to explore these relationships and draw inferences about the underlying processes.

Eigendecomposition derives a unique set of perpendicular vectors that represent the relationships between electrode pairs sorted according to variance, and an associated set of weights for the relative contribution of each vector to the covariance matrix. Eigendecomposition of a covariance matrix is the fundamental step in principal components analysis (PCA), where measured data are projected into a new coordinate system that maximize variation in the data. Generalized eigendecomposition (GED) extends this concept of sorting by variance and offers comparative analysis between exposure conditions. The difference in variance between conditions is calculated and mapped back to the original channel locations where it is assumed that greater change reflects brain involvement between conditions (26).

## Results

Six children were approached, recruited, and completed participation in the experimental session (*n* = 6). Target recruitment of *n* = 10 was not achieved due to COVID-19 related reductions in non-emergency admission at RCH during the study period. The basic demographic and diagnostic information, and details of the experimental session are included in Table 1.

**Table 1:** Participant demographic and diagnostic data.

Participant	Age	Sex	Handedness	Diagnosis	Experimental session
1	8yr 7months	Female	Right dominant	Tuberous Sclerosis Focal Epilepsy Mild language delay	Song—Speech—Noise
2	5yrs 0months	Female	Right dominant	Left hippocampal Sclerosis Focal Epilepsy	Noise—Speech—Song
3	8yrs 11months	Male	Right dominant	Focal Epilepsy ADHD	Song—Speech—Noise
4	2yrs 9months	Female	Right dominant	? Absence seizures Nil other	Speech—Noise—Song
5	19months	Male	NA	Tuberous Sclerosis Tuber in L angular region Focal seizures	Speech—Noise—Song
6	8yrs 7months	Male	Left dominant	ASD—high functioning Epilepsy- infrequent seizures	Song—Noise—Speech

**Figure 3:**
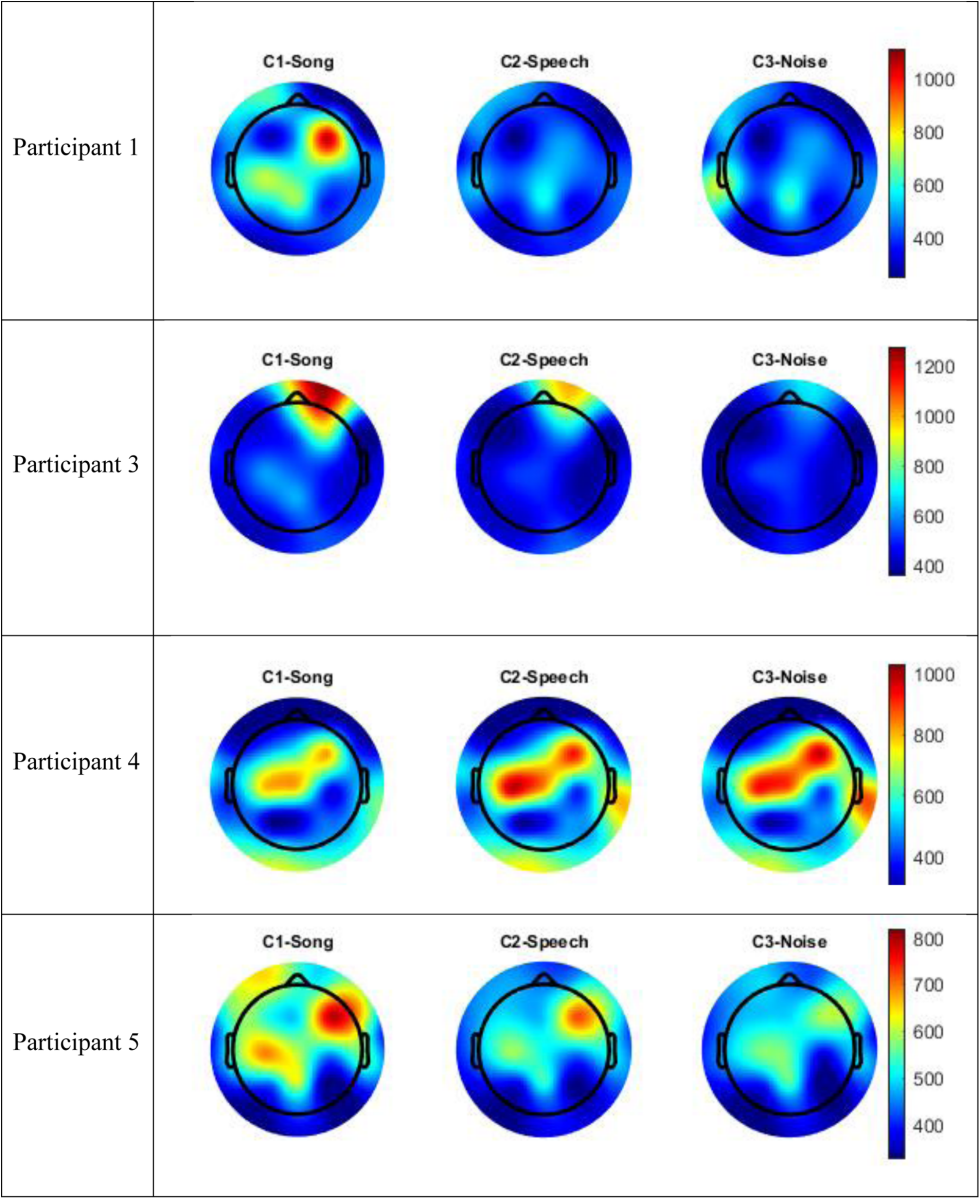
Topographic maps mean amplitude broadband frequency range (0.5–40 Hz) per condition compared to silence.

**Figure 4:**
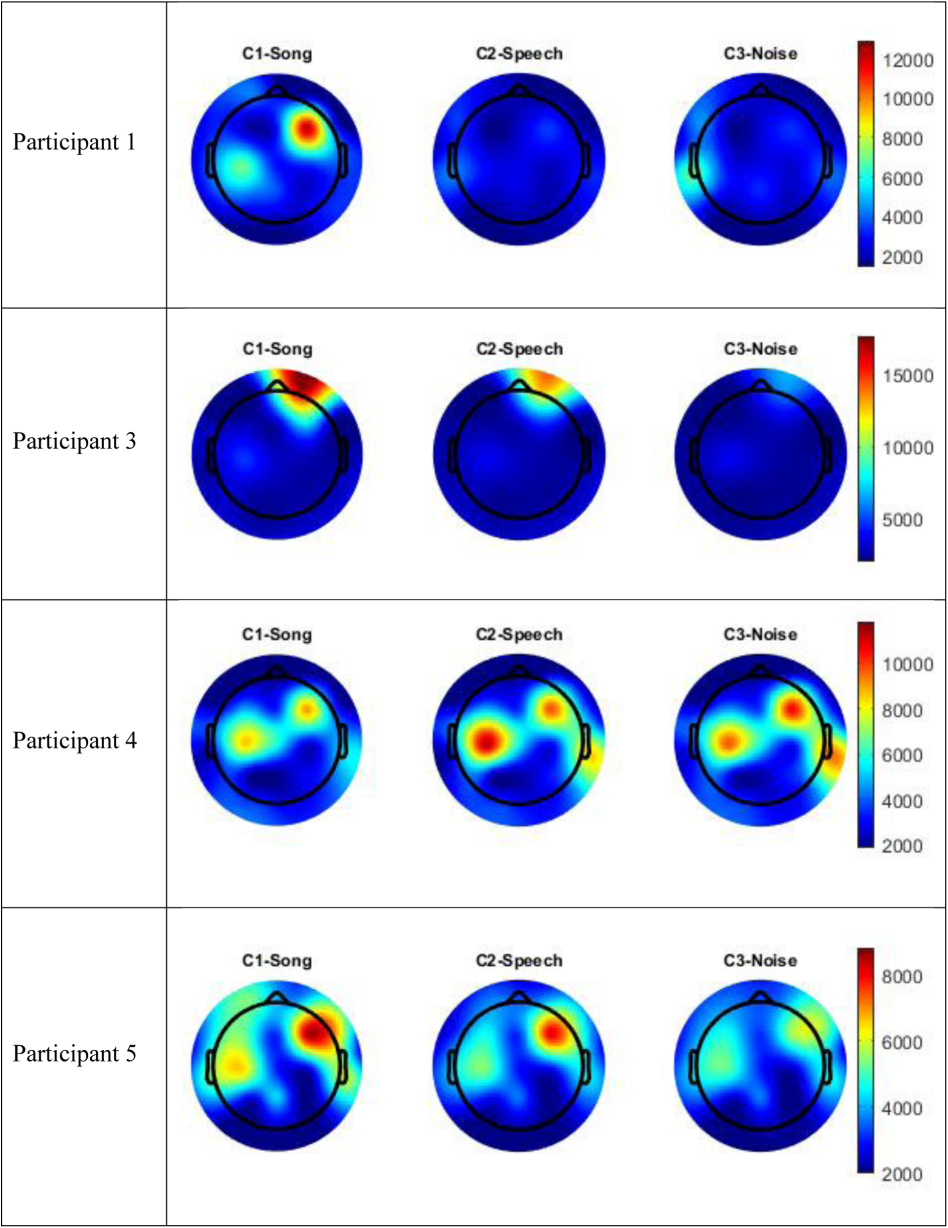
Topographic maps mean amplitude Beta frequency range (13–30 Hz) per condition compared to silence.

The data for Participants 2 and 6 was excluded from analysis. A technical error occurred with the transfer of data from the clinical system for Participant 2, and Participant 6 interacted with the iPad by playing a game during the experimental session meaning their state was not controlled. Therefore, only the data for four participants (1, 3–5) were included in the analysis. The following results of the frequency analysis include the topographic maps for the four participants of the mean amplitude of the broadband frequency range (0.5–40 Hz) in Figure 3, and the frequency analysis of the Beta range (13–30 Hz) in Figure 4.

Visual inspection of the spectral components suggests there are some changes in underlying frequency components in the song condition that are unique to this condition. Participants 1 and 5 recorded an increase energy in the EEG signal (increased mean amplitude) in the right frontal-temporal leads in both the broadband frequency range and the Beta frequency range, and this was greater than recorded in either the speech or noise conditions. This increase in energy is indicated in red in the topographic maps. Participant 3 also recorded an increase in mean amplitude during the song condition in the right frontal leads in both the broadband frequency range and the Beta frequency range, and this was greater than either the speech or noise condition. Participant 4 recorded a paradoxical response and had a decrease in EEG signal in the fronto-central regions in both the broadband and delta frequency ranges during the song condition, compared to the speech or noise conditions. Nonetheless, in all four participants, there were changes in underlying frequency components in the song condition that were unique to that condition; that is, different to what was observed in the speech or noise conditions.

**Figure 5:**
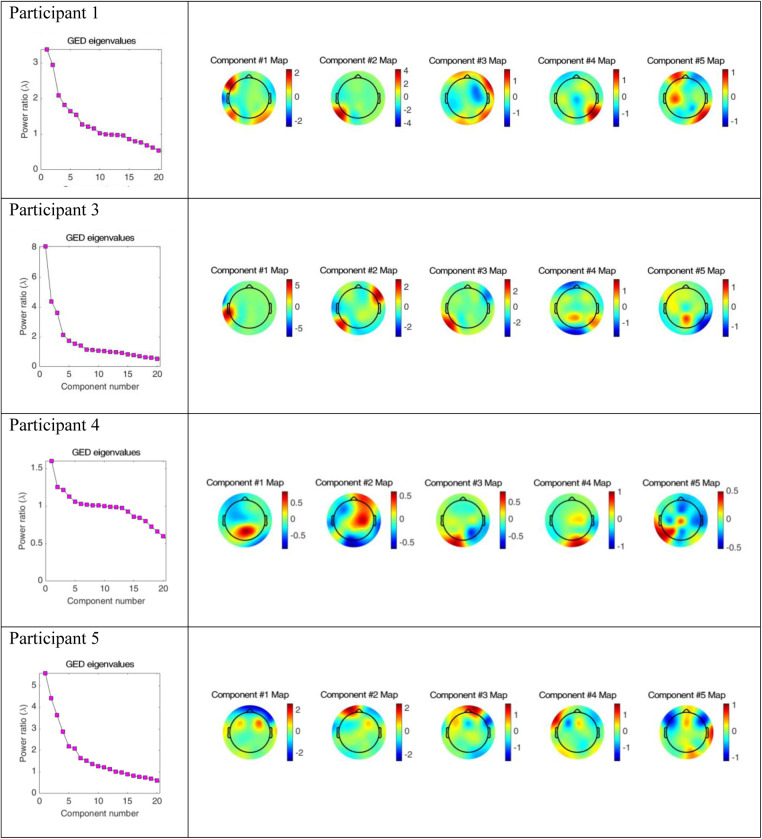
Scree plots and component topographic maps of song vs. silence for the top five components of the EEG signal for each of the four participants.

Figure 5 includes the analysis of variance (GED) for the song conditions vs. the baseline silence for each participant. Both the scree plots and component topographic maps are included. The topographic maps include the first five components for each participant, that is the components with the highest covariance. The GED analysis did not yield a response that was reproducible across all participants. That is, the mean amplitude changes recorded in the EEG signal in the broadband and Beta frequency ranges (Figures 3, 4) did not correlate with consistent topographical patterns of variance across the four participants.

## Discussion

This study demonstrates the feasibility of collecting EEG data in a clinical setting that is sufficient to identify a unique response to song in children, compared to speech and noise. Despite the imperfect auditory environment of an acute hospital, power spectrum analysis revealed distinct changes in frequency components during the song condition, which were unique to that condition. Participants 1, 3, and 5 exhibited increased EEG signals within the broadband frequency range during the song condition, underscoring the potential of this data collection method to enhance our understanding of how children process music. Therefore, utilizing a pre-existing clinical EEG offers significant promise for advancing music neuroscience research in the underrepresented pediatric population.

One hundred percent of children approached were recruited for this study and completed participation in the experimental session. This type of research was arguably low burden for participants and their families, and the utilisation of pre-existing clinical EEG affords significant potential for the expansion of knowledge with applications in real world settings. Further, the data collected via the clinical EEG system was high quality data. The independent component analysis (ICA) identified only two artifacts for removal in the EEG data: eye blink and muscle movements. These artifacts are well recognized artifacts in EEG data, and it is notable that no line noise was identified for removal. The study further supports arguments that EEG is a highly practical method of brain scanning for use with children given the high tolerance for movement (18).

The GED method of EEG analysis is a uniquely hypothesis driven multivariate statistical analysis method that has not been commonly employed in music-based neuroscience research. Spectral analysis of EEG data seeks to localize complex brain responses to single electrodes via a univariate statistical analysis; the GED method extends this analysis. Traditionally, a critique of EEG has been the relatively poor spatial localization compared to hemodynamic methods like fMRI (28). However, the GED method of EEG analysis was utilized in this study because multivariate statistical analysis increases the potential for a more statistically reliable source localization during complex cognitive tasks (26). The neural circuits involved in complex cognitive tasks, like the processing of song, are hypothesized to be combinations of functional networks of neurons that generate widespread voltage changes recorded by multiple scalp electrodes simultaneously. Thus the GED analysis considers that the signals responsible for musical processing likely span many channels of the EEG and facilitates the isolation and extraction of information across these channels (26). That is, the GED method acknowledges that it is not feasible for a one-to-one mapping of electrode to computational source and extends current uses of EEG in music neuroscience research to incorporate more complex whole music stimuli.

The implementation of the GED method of EEG analysis did not isolate a source responsible for this observed change across the participants in this study. While there was a different EEG signal recorded in the participants during the song condition, it was not possible to isolate neural generators of this response that were consistent across the participants. Based on visual inspection, the mean amplitude shown in Figures 3, 4 above did not correlate with covariance plots in Figure 5. This was not an unexpected finding given the age range of participants, the small number of participants and the small number of iterations of each condition for participants. Thus, based on the data collected in this study, the null hypothesis was accepted. The acceptance of the null hypothesis in this feasibility study does not negate the potential value of the GED method in music neuroscience research, rather modifications should be made to the study design relating to data collection. Philosophically, it is also possible that increased energy on the mean amplitude topographic maps may lead to decreased co-variance. This is indicated as a cold spot on the component topographic maps due to an increase in synchronous activity during music and the phenomena of super-position cancellation that would lead to variance tending towards zero. However, this remains purely speculative related to this study, and further research is necessary.

It is fundamental to note that the GED analysis not locating a consistent signal source across the participants is not the same as the individual participants not having a response to, or an experience of, the song condition. Rather, that each participant did record a unique EEG signal during the song condition, however, the neural generators of this response were not consistent across the participants. It is reasonable to extrapolate that for Participants 1, 3, and 5, under the experimental conditions utilized in this study, music was a richer sensory experience that stimulated a larger brain response than either the speech or noise conditions. The localization of the increased mean amplitude to the right hemisphere aligns with current models of music processing in adults, however, a larger activation in the temporal regions may be expected based on these adult models (29). Participant 4 recorded a different response to the song condition compared to the speech or noise conditions, the response was a decrease in mean amplitude in the central and right frontal leads. Based on the data collected in this study, it is not possible to explain why Participant 4 appeared to have the opposite response to familiar song to the other three participants.

The children recruited for participation in this feasibility study all had a clinical indication for EEG monitoring with some history of seizures/suspected seizures, even if infrequently. Therefore, these participants may not have been a suitable control cohort if the intention of the study had been comparative. Arguably, this did not impact the outcomes of the study given the pragmatic intention to explore the feasibility of the clinical methodology.

The larger EEG signal recorded in three participants during the song condition also suggest significant potential for music-based interventions in clinical settings. Music may be more effective than other auditory stimuli in activating cortically mediated responses in children (30). Conversely, it is essential to consider the possibility that exposure to music in neurologically vulnerable populations may result in cognitive fatigue and overstimulation. These promising clinical applications require rigorous scientific exploration.

## Study limitations

All interpretation of the results of this study should be contextualized within the feasibility design. The intention of this study was not to draw population inferences related to song processing in children or the developmental trajectory of the neural response to whole music, rather determine the feasibility of collecting data in the clinical setting that may support future statistically powered studies. The impracticalities and research burden of having neurotypical healthy children attend an acute healthcare setting solely for research purposes likely contributes to the underrepresentation of children in neuroscience research. Unfortunately, during the recruitment period for this study a pause was placed on all non-emergency hospital admissions in anticipation of an influx of COVD-19 related hospital admissions, and this further reduced the number of children who could be recruited. Given time constraints of the study, it was not possible to extend data collection.

The comparatively wide age range of the participants was a significant additional variable that resulted in limitations in the study and difficulties with generalizability of the results. All data analysis was undertaken on a single participant level and it is therefore beyond the scope of this study to explore any changes in processing across age cohorts related to brain maturation, or to hypothesize any impact of typical or non-typical neural developmental on the processing of the song. Previous research has noted the immaturity of cortical auditory pathway in children, resulting in delayed latency and variability in the localization of components of the evoked response (31). Based on this evidence, it is reasonable to extrapolate a delayed maturation of brain response to the complex stimulus of familiar song and comparative research across various age cohorts is essential to further explore this maturation process.

Neuroscience research has the potential to support an understanding of brain processing of music, functional networks employed during music tasks, and changes in brain structure/neuroplasticity that may be stimulated by engagement in music (32). However, a limitation of traditional EEG research is that it is implausible to reduce complex musical experiences to measurable electrical activity that can be recorded at the scalp (18). While this study may have expanded current knowledge with the inclusion of a more ecologically valid music experience and multivariate statistical analysis of the EEG data, the GED method relies on multiple assumptions. These assumptions include that differences in variance reflect different brain activity related to auditory conditions (music compared to silence). It is possible that the changes might also be the result of unrelated brain states or differences in measurement noise. Future research may also seek to test GED against other multivariate statistical methods and haemodynamic methods of brain scanning to localise source generation. It also remains that music listening encapsulates the rich interplay of cognitive and emotional processing, entwined with the immeasurable cultural and social heritage of the individual child and it was beyond the scope of this study to explore this phenomenon.

### Considerations for future research

Having established the feasibility of the method for its intended purpose, future research should seek to develop appropriately powered studies to track the development of responses to whole music, to developed greater knowledge of source localization. Further adjustments are also needed in future research to increase the utility of the results. Implementing a more restrictive study design may decrease the signal to noise ration to improve the likelihood of isolating a unique and reproducible response in children. This may include controlling for potential developmental differences by recruiting to specific age cohorts (e.g., 1–2 year, 3–5 years etc), and utilising a simple novel repetitive moving graphic to offer greater control of state via visually engaging the participants without emotional content of a familiar movie.

Increasing the number of iterations of each auditory condition for participants will also reduce the signal to noise ration of the EEG data. Experimental sessions were intentionally short in this feasibility study as acceptability of the research design had not previously been explored with children who are awake/conscious. However, future research could seek to extend this duration.

Potentially the most important consideration for future research is to increase the number of participants. Once baseline knowledge of a unique EEG response to song and generators of this response are described in neurotypical children, the methodology could be applied to clinical populations to understand organic and non-organic (e.g., pharmacological) induced changes in music processing in clinical populations. This has the potential to support a mechanistic understanding of music-based/music therapy interventions or even support screening for developmental delays in populations where standard testing is difficult. The method of EEG data collection and analysis utilized in this study could also be extended to clinical populations where EEG is employed to describe responses to music in the absence of reliable behavioural indicators, for example children presenting with reduced consciousness.

## Conclusion

EEG has enormous potential for capturing data about brain states, in real world scenarios, to increase the clinical translatability of music neuroscience knowledge for clinical applications of music within acute healthcare. This study demonstrated feasibility of collecting useful data via a pre-existing clinical system to describe the processing of an ecologically valid stimulus. Collecting data via a clinical system affords a pragmatic yet innovative opportunity to expand knowledge in the highly underrepresented pediatric population. This study design also supports the exciting potential for ongoing collaborations between clinicians and scientist to ensure knowledge is relevant and translatable to real world settings and could potentially be extended to other clinical methods of brain scanning including MRI/fMRI. The novel application of a GED analysis in this study progressed current knowledge of music processing in children beyond the time-frequency domain. This multivariate approach supports the ability to isolate and extract information that is distributed across electrodes (26). The use of a GED supports the source localization of (whole) song, as an ecologically valid music experience compared to pure tones or similarly deconstructed musical stimuli common in auditory neurosciences. While we were not able to isolate a consistent neural source of the response across the participants, data from the individual participants indicates that familiar song stimulated a unique brain response, compared to the speech or noise conditions. Further, results have potential implications for clinical applications of music because EEG may be used to describe a covert response to music in the absence of a reliable behavioural indicator. Additional research should seek to reduce variation among participants via greater recruitment and control of age cohorts and increase the iterations of the auditory conditions to decrease the signal to noise ratio.

## Data Availability

The datasets presented in this article are not readily available due to the HREC requirement for ethical approval, which is that: de-identified EEG data may be available for use by future researchers from a recognized research institution whose proposed use of the data has been ethically reviewed and approved by an independent committee and who accept MCRI’s conditions for access. Request to access the datasets should be directed to the corresponding author.
